# Supplementary material for: Plasmodium falciparum PP1 phosphatase is a key regulator of malaria parasite transmission
Source: mBio. 2025 Aug 18;16(9):e00874-25. doi: 10.1128/mbio.00874-25 (PMC12421956; doi:10.1128/mbio.00874-25)
Supplement: Supplemental material — Supplemental tables and figures. [file mbio.00874-25-s0001.pdf]

Plasmodium falciparum PP1 phosphatase is a key regulator of malaria parasite transmission

Royer Ludivine[1#], Colard-Itté Emma[1#], Tavella Tatyana[1], Lorthiois Audrey[2], Goussin Stéphane[2], N'Dri Marie-Esther[1], Sabra Reem[3], Auréline Deiss[1], Thiberge Sabine[2], Lamarque Mauld H.[3], Lavazec Catherine[1]*

Supplemental Material

Gene ID	Symbol	name	Superfamily	TPM Ring	TPM Early troph	TPM Late troph	TPM Schizont	TPM Gam Stage II	TPM Gam Stage V
PF3D7_1414400	PP1	serine/threonine protein phosphatase PP1	MPP (Metallophosphatase)	22,21	26,86	170,82	90,06	50,23	195,76
PF3D7_0925400	PP2	protein phosphatase beta	MPP (Metallophosphatase)	6,7	6,46	14,84	3,3	17,77	23,7
PF3D7_0802800	PP3	serine/threonine protein phosphatase 2B	MPP (Metallophosphatase)	7,9	0,83	56,59	68,85	6,19	17,21
PF3D7_0927700	PPP4	serine/threonine protein phosphatase 4	MPP (Metallophosphatase)	25,12	1,85	20,23	7,11	24,79	39,82
PF3D7_1355500	PP5	serine/threonine protein phosphatase 5	MPP (Metallophosphatase)	28,96	14,37	15,6	1,28	2,91	14,76
PF3D7_0314400	PPP6	serine/threonine protein phosphatase 6	MPP (Metallophosphatase)	36,11	11,51	11,02	1,99	15,63	25,87
PF3D7_1423300	PP7	serine/threonine protein phosphatase 7	MPP (Metallophosphatase)	9,59	0,78	35,06	25,96	11,42	57,78
PF3D7_1018200	PPP8	pseudophosphatase PPP8	MPP (Metallophosphatase)	16,4	1,78	46,07	79,3	6,66	48,53
PF3D7_1469200	SHLP1	shewanella-like protein phosphatase 1	MPP (Metallophosphatase)	8,82	0,84	5,64	1,91	7,59	99,43
PF3D7_1206000	SHLP2	shewanella-like protein phosphatase 2	MPP (Metallophosphatase)	5,7	0,45	9,41	7,59	4,43	25,31
PF3D7_1466100	PPKL	protein phosphatase containing kelch-like domains	MPP (Metallophosphatase)	6	1,23	19,81	8,18	5,86	18,82
PF3D7_0410300	PPM1	protein phosphatase PPM1	PP2Cc (Protein phosphatases 2c domain)	26,85	12,52	12,51	3,32	3,59	9,1
PF3D7_1138500	PPM2	protein phosphatase PPM2	PP2Cc (Protein phosphatases 2c domain)	138,71	30,49	78,17	16,89	10,96	57,72
PF3D7_1455000	PPM3	protein phosphatase PPM3	PP2Cc (Protein phosphatases 2c domain)	4,78	1,51	1,09	0,7	3,77	24,36
PF3D7_1249300	PPM4	protein phosphatase PPM4	PP2Cc (Protein phosphatases 2c domain)	29,4	13,49	25,15	4,69	2,7	11,17
PF3D7_0810300	PPM5	protein phosphatase PPM5	PP2Cc (Protein phosphatases 2c domain)	10,73	13,05	70,65	31,75	22,62	85,93
PF3D7_1309200	PPM6	protein phosphatase PPM6	PP2Cc (Protein phosphatases 2c domain)	84,23	41,83	36,64	14,33	10,74	30,51
PF3D7_0810500	PPM7	protein phosphatase PPM7	PP2Cc (Protein phosphatases 2c domain)	152,34	45,45	29,37	6,93	24,26	81,02
PF3D7_1135100	PPM8	protein phosphatase PPM8	PP2Cc (Protein phosphatases 2c domain)	61,84	12,93	21,6	13,23	3,33	51,75
PF3D7_0520100	PPM9	protein phosphatase PPM9	PP2Cc (Protein phosphatases 2c domain)	29,88	11,77	6,9	1,29	3,37	8,77
PF3D7_1009600	PPM10	protein phosphatase PPM10	PP2Cc (Protein phosphatases 2c domain)	7,86	15,59	8,98	0,24	2,9	22,25
PF3D7_1455100	PTP1	protein tyrosine phosphatase	PTPc (Protein Tyrosine phosphatase)	70,47	109,92	21,66	2,18	21,31	121,37
PF3D7_1113100	PRL	protein tyrosine phosphatase	PTPc (Protein Tyrosine phosphatase)	170,84	84,04	41,71	18,3	31,34	105,84
PF3D7_0309000	YVH1	dual specificity protein phosphatase	PTPc (Protein Tyrosine phosphatase)	60,42	9,65	11,08	2,1	6,34	30,8
PF3D7_0515900	NIF2	NLI interacting factor-like phosphatase	HAD_like (Haloacid Dehalogenase)	6,47	14,3	15,06	6,44	4,9	44,22
PF3D7_1355700	NIF3	NLI interacting factor-like phosphatase	HAD_like (Haloacid Dehalogenase)	63,86	32,67	22,22	5,36	8,66	42,63
PF3D7_1012700	NIF4	NLI interacting factor-like phosphatase	HAD_like (Haloacid Dehalogenase)	16,21	6,12	11,28	2,63	2,17	7,39
PF3D7_0726900	TIM50	mitochondrial import inner membrane translocase subunit	HAD_like (Haloacid Dehalogenase)	7,8	9,49	8,01	0,7	1,63	19,57

Supplemental Table 1. *Plasmodium falciparum* phosphatome.

The *P. falciparum* phosphatome as reported in Guttery *et al*, 2014, and transcription profiles of each phosphatase during different *P. falciparum* life stages reported in Lopez-Barragan et al, 2011 (https://plasmodb.org/). The 12 phosphatases more highly transcribed in sexual stages than in asexual stages are underline in blue. TPM: Transcripts per Million. troph: trophozoites.

Transmission parameters	Experiment 1 NF54	Experiment 1 -RAP	Experiment 1 +RAP	Experiment 2 NF54	Experiment 2 -RAP	Experiment 2 +RAP	Experiment 3 NF54	Experiment 3 -RAP	Experiment 3 +RAP
n (no. of mosquitoes scored)	28	45	48	18	49	44	20	35	36
Oocyst prevalence (%)	96%	24%	13%	89%	24%	11%	90%	34%	28%
Median oocyst density (no. of oocysts per mosquito)	38	0	0	16.5	0	0	81.5	0	0
Mean oocyst density	55.9	1.5	1.4	20.0	2.3	2.2	86.1	2.1	1.9
range	0 - 222	0 - 2	0 - 2	0 -44	0 - 5	0 - 4	0 - 190	0 - 5	0 - 7

Supplemental Table 2. Transmission parameters of *Pf*PP1-depleted parasites to Anopheles mosquitoes

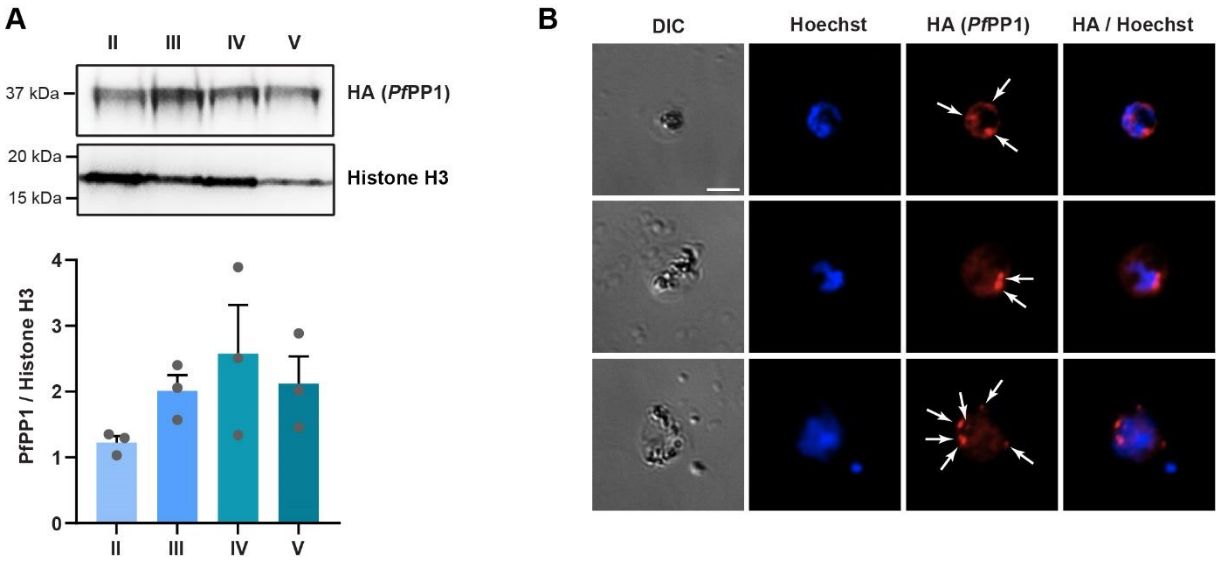

Supplemental Figure 1. Expression and localization of *Pf*PP1.

A. Immunoblot analysis showing expression of *Pf*PP1-HA in different gametocyte stages using anti-HA antibodies. Histone H3 protein was used as a loading control. Protein lysates derived from an equal number of parasites were loaded per lane. Quantification of *Pf*PP1 levels relative to Histone H3 (lower panel) was performed by densitometry (Quantity one software). **B.** Immunofluorescence analysis of *Pf*PP1 during mitosis in male gametes. Representative images of activated male gametes co-labeled with anti-HA antibody and Hoechst. *Pf*PP1 is localized in 2 to 8 foci associated with the dividing nucleus in male gametes. DIC: Differential Interference Contrast. Scale bars: 2µm.

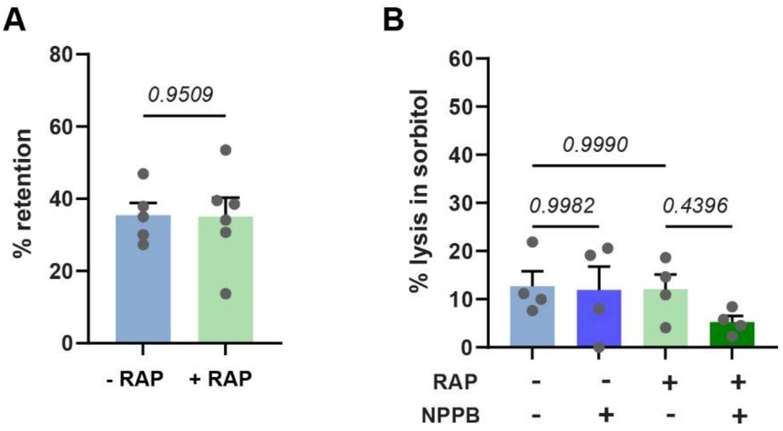

Supplemental Figure 2. Rapamycin treatment does not affect GIE mechanical properties in wild-type parasites

A. Retention in microbeads of stages V GIE from the B10 clone treated with DMSO (-RAP) or with rapamycin (+RAP). Bars represent the mean ± SEM from 2 independent experiments in 3 technical triplicates (n = 3). Statistical analyses were performed using a Mann-Whitney test.

B. Sorbitol-induced isosmotic lysis of stages V GIE from the B10 clone treated with DMSO (-RAP) or with rapamycin (+RAP), in the presence or absence of 100 µM NPPB. Bars represent the mean ± SEM from 2 independent experiments in 2 technical replicates (n = 2). Statistical analyses were performed using a one-way ANOVA test.

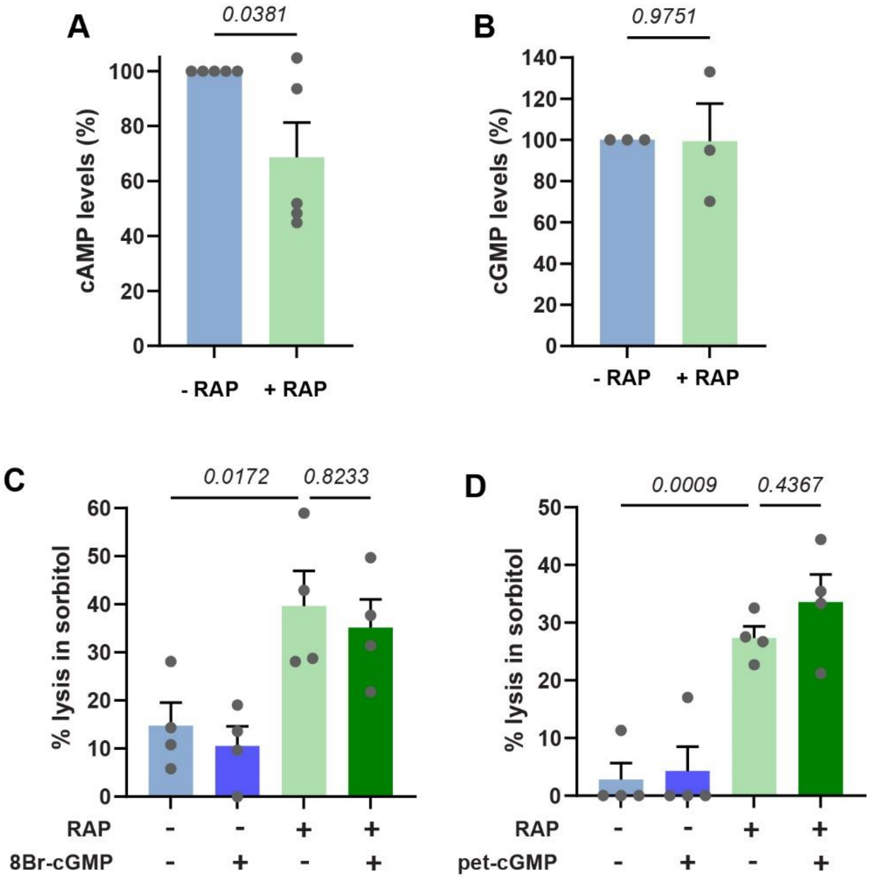

Supplemental Figure 3. *Pf*PP1 does not control GIE mechanical properties through modulation of cyclic nucleotide levels

A-B. Relative cAMP (A) and cGMP (B) levels in stage V GIE from the *pfpp1-iKO* transgenic line treated with DMSO (-RAP) or with rapamycin (+RAP). Bars represent the mean ± SEM from 5 independent experiments (n = 5) for cAMP levels and 3 independent experiments (n = 3) for cGMP levels. Statistical analyses were performed by unpaired t-test. **C-D.** Sorbitol-induced isosmotic lysis of stages V GIE from the *pfpp1-iKO* transgenic line treated with DMSO (-RAP) or with rapamycin (+RAP), supplemented or not with 1 µM 8Br-cGMP (C) or 1 µM pet-cGMP (D). Bars represent the mean ± SEM from 4 independent experiments (n = 4). Statistical analyses were performed by one-way ANOVA test.

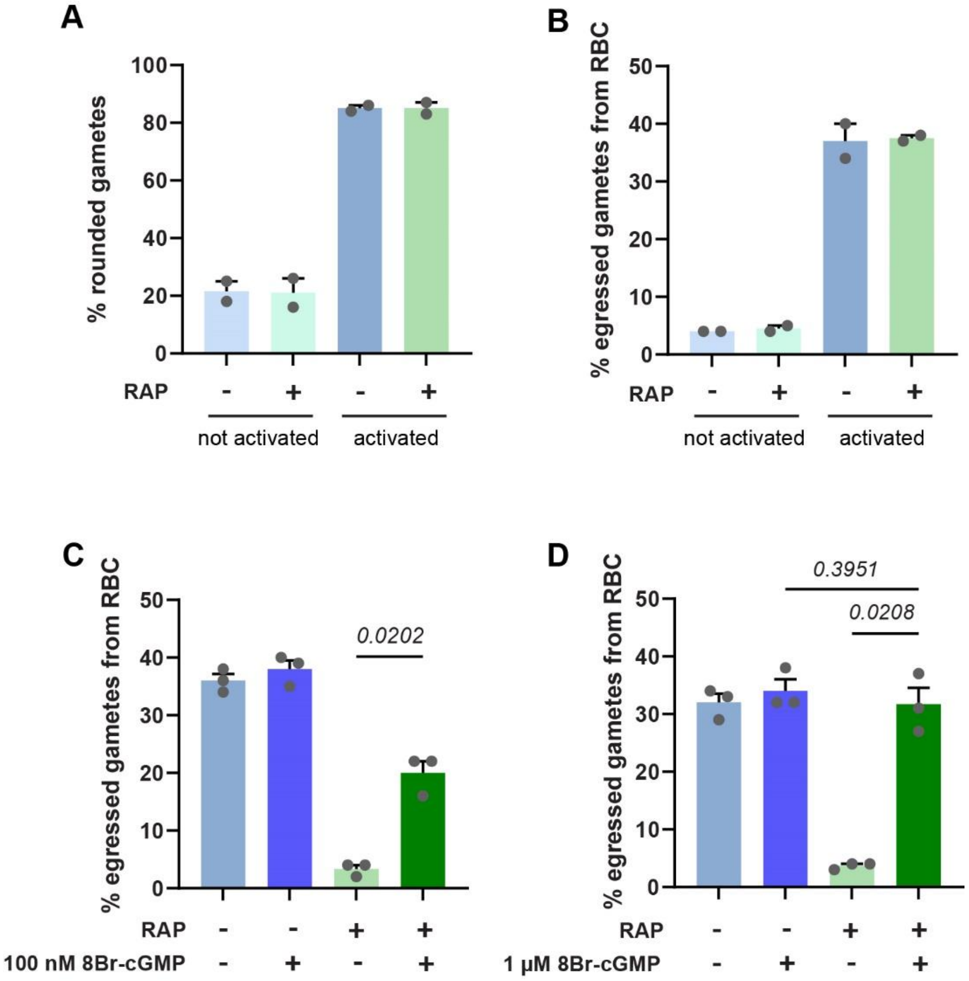

Supplemental Figure 4. Rapamycin treatment does not affect gametogenesis in wild-type parasites

A-B. Percentage of gametocyte rounding up (A) and egressed gametes from the erythrocyte (B) before activation (not activated) and 20 min following activation (activated) of stage V GIE from the B10 clone treated with DMSO (-RAP) or with rapamycin (+RAP). Bars represent the mean ± SEM from 2 independent experiments (n = 2). **C-D.** Percentage of egressed gametes from the erythrocyte before and after activation of stage V GIE from the *pfpp1-iKO* transgenic line treated with DMSO (-RAP) or rapamycin (+RAP), supplemented or not with 100 nM (C) or 1µM (D) 8Br-cGMP. Bars represent the mean ± SEM from 3 independent experiments (n = 3). Statistical analyses were performed by one-way ANOVA test.
